# Characterization of Two Metal Binding Lipoproteins as Vaccine Candidates for Enterococcal Infections

**DOI:** 10.1371/journal.pone.0136625

**Published:** 2015-08-31

**Authors:** Felipe Romero-Saavedra, Diana Laverde, Aurélie Budin-Verneuil, Cécile Muller, Benoit Bernay, Abdellah Benachour, Axel Hartke, Johannes Huebner

**Affiliations:** 1 Division of Infectious Diseases, Department of Medicine, University Medical Center Freiburg, Freiburg, Germany; 2 EA4655 U2RM Stress/Virulence, University of Caen Lower-Normandy, Caen, France; 3 Division of Pediatric Infectious Diseases, Dr. von Hauner Children's Hospital, Ludwig-Maximilians-University, Munich, Germany; 4 Proteogen platform SFR ICORE 4206, University of Caen Lower-Normandy, Caen, France; University of Illinois at Chicago College of Medicine, UNITED STATES

## Abstract

**Background:**

*Enterococcus faecium* and *faecalis* are Gram-positive opportunistic pathogens that have become leading causes of nosocomial infections over the last decades. Especially multidrug resistant enterococci have become a challenging clinical problem worldwide. Therefore, new treatment options are needed and the identification of alternative targets for vaccine development has emerged as a feasible alternative to fight the infections caused by these pathogens.

**Results:**

We extrapolate the transcriptomic data from a mice peritonitis infection model in *E*. *faecalis* to identify putative up-regulated surface proteins under infection conditions in *E*. *faecium*. After the bionformatic analyses two metal binding lipoproteins were identified to have a high homology (>72%) between the two species, the manganese ABC transporter substrate-binding lipoprotein (PsaA_fm,_) and the zinc ABC transporter substrate-binding lipoprotein (AdcA_fm_). These candidate lipoproteins were overexpressed in *Escherichia coli* and purified. The recombinant proteins were used to produce rabbit polyclonal antibodies that were able to induce specific opsonic antibodies that mediated killing of the homologous strain *E*. *faecium* E155 as well as clinical strains *E*. *faecium* E1162, *Enterococcus faecalis* 12030, type 2 and type 5. Mice were passively immunized with the antibodies raised against recombinant lipoproteins, showing significant reduction of colony counts in mice livers after the bacterial challenge and demonstrating the efficacy of these metal binding lipoproteins as promising vaccine candidates to treat infections caused by these enterococcal pathogens.

**Conclusion:**

Overall, our results demonstrate that these two metal binding lipoproteins elicited specific, opsonic and protective antibodies, with an extensive cross-reactivity and serotype-independent coverage among these two important nocosomial pathogens. Pointing these two protein antigens as promising immunogens, that can be used as single components or as carrier proteins together with polysaccharide antigens in vaccine development against enterococcal infections.

## Introduction

Enterococci are normal inhabitants of the gastrointestinal tract of animals and humans, but have been reported also as causative agent of infectious diseases in humans [1]. In recent years enterococci have emerged as important nosocomial pathogens due to their multiple antibiotic resistances, ranking *E*. *faecalis* and *E*. *faecium* as the third and fourth most commonly isolated species [2–5]. Until 1980s, the majority of enterococcal infections were caused by *E*. *faecalis*, but since the beginning of 1990s *E*. *faecium* has become as common as cause of nosocomial infections as *E*. *faecalis* [6,7]. This shift in enterococcal epidemiology may be due to the high levels of antibiotic resistance that *E*. *faecium* presents in contrast to *E*. *faecalis* [6]. Therefore, there is an urgent need to develop alternative therapies and preventive strategies against enterococcal infections [8,9]. Currently, vaccines and immunotherapies are among the most promising alternative approaches to fight these opportunistic pathogens, since they allow specific targeting, not affecting commensal flora, and therefore are associated with a low risk of development of bacterial resistance [10].

In Gram-positive bacteria, lipoproteins are involved in many important cellular processes within the subcellular region of the cell envelope between the plasma membrane and the outer layers of the cell (i.e. peptidoglycan and other layers of the cell wall). Molecules residing in the area represent approximately 2–3% of the bacterial proteome [11,12]. The most abundant functional group of lipoproteins are substrate binding proteins (SBPs) which deliver substrate-binding proteins to ATP-binding cassette (ABC) transporters, accounting for ~40% of the predicted lipoproteins. ABC transporters are classified into at least nine subfamilies according to the substrate transported [12]. Lipoproteins perform diverse functions including nutrient and substrate uptake, folding of excreted proteins, conjugation, antibiotic resistance and transport [11,13]. In Gram-positive bacteria, some lipoproteins have been demonstrated to play crucial roles in host-pathogen interactions such as adhesion, colonization and initiation of inflammatory processes by recruiting immune cells and activating toll-like receptor 2 [11–14]. To date, many lipoproteins from several bacterial pathogens, as well as the proteins and enzymes involved in their biosynthesis, have been studied and proposed as potential vaccine candidates and targets for drug development [13,14]. The rationale behind a lipoprotein-directed vaccine relies in the immunostimulatory activity, specific location and the potential implication in virulence that these protein-antigens possess [11,12].

Few studies have been conducted to determine the role of lipoproteins in enterococcal virulence. Rince and co-workers identified lipoprotein-encoding genes in the genome of the clinical isolate *E*. *faecalis* V583 and analyzed their putative function. Among the predicted lipoproteins, 43% accounted as components of the ABC transporters and 40% have been already demonstrated either to be involved in *E*. *faecalis* virulence or to share high homologies with lipoproteins implicated in virulence of other Gram-positive pathogens [11]. The prolipoprotein diacylglyceryl transferase (*Lgt*) and the *E*. *faecalis* antigen A (*efaA*) lipoproteins have been described to be involved in stress response and virulence [14,15]. Until now, the potential use of these proteins as vaccine candidates in enterococcus has not been explored. Interestingly, Burnie and co-workers examined antibody responses in sera from patients infected by vancomycin-resistant *E*. *faecium*, and demonstrated that one phage antibody, directed against amino acid sequences containing ABC transporters, was able to reduce colony counts in a mouse infection model [16]. In the present study, the transcriptomic data obtained from a bacteremia mouse model with *E*. *faecalis* was used to identify putative cell-wall related lipoproteins with high homologies in the vancomycin-resistant *E*. *faecium* E155. The putative *in vivo* up regulated and cell-wall related proteins were overexpressed in *E*. *coli*, purified and immunologically confirmed to be potential protein vaccine candidates against enterococcal infections.

## Materials and Methods

### Bacterial strains and sera

The bacterial strains and sera used for the present study are listed in the Table 1 [17–21]. Polyclonal sera against the recombinant proteins were produced as follows: New Zealand white rabbits were immunized with two subcutaneous injections of 10 μg protein given 2 weeks apart; in the third week, three injections of 5 μg were given intravenously every other day. Finally, in the fifth week two injections of 5 μg were given intravenously with three days between doses. Three different sera were obtained from each rabbit: a pre-immune serum seven days prior to the first immunization (Day 0, NRS-protein), a test bleed serum 15 days after the fifth immunization (Day 45, Test-protein) and a terminal bleed serum collected five days after the last immunization (Day 63, Anti-protein). All sera were heat inactivated at 56°C for 30min and frozen at -20 C.

**Table 1:** Bacterial strains and sera used for this study.

Strain or serum	Description^*^	Reference or source
**Strains**		
***E*. *faecium* E155**	ARE, VRE strain isolated from a patient in the USA (Chicago), CC17	[17]
***E*. *faecium* E1162**	ARE strain isolated from blood in the Netherlands, CC17	[18]
***E*. *faecalis* 12030**	isolated from a patient in the USA (Cleveland)	[19]
***E*. *faecalis* type 2**	isolated from a patient in Japan (Sapporo)	[20]
***E*. *faecalis* type 5**	isolated from a patient in Japan (Kobe)	[20]
***E*. *coli* M15pRep4**	M15 harboring pRep4 plasmid	(INVITROGEN)
***E*. *coli M15/*pQE30AdcA**_**fm**_	M15 harboring pRep4 and pQE30AdcA_fm_ plasmids	This study
***E*. *coli M15/*pQE30PsaA**_**fm**_	M15 harboring pRep4 and pQE30PsaA_fm_ plasmids	This study
**Sera**		
**NRS**	Pre-immune sera from rabbit	This study
**Anti-SagA**	Rabbit serum raised against the recombinant SagA	[21]
**NRS-AdcA**_**fm**_	Pre-immune sera from rabbit immunized with AdcA_fm_ collected at day 0	This study
**Test-AdcA**_**fm**_	Rabbit serum raised against the recombinant AdcA_fm_ collected at day 45	This study
**Anti-AdcA**_**fm**_	Rabbit serum raised against the recombinant AdcA_fm_ collected at day 63	This study
**NRS-PsaA**_**fm**_	Pre-immune sera from rabbit immunized with PsaA_fm_ collected at day 0	This study
**Test-PsaA**_**fm**_	Rabbit serum raised against the recombinant PsaA_fm_ collected at day 45	This study
**Anti-PsaA**_**fm**_	Rabbit serum raised against the recombinant PsaA_fm_ collected at day 63	This study

^*^ AdcA_fm_, zinc ABC transporter substrate-binding lipoprotein from *E*. *faecium*; ARE, ampicillin resistant enterococci; CC17, clonal linage complex 17; PsaA_fm_, manganese ABC transporter substrate-binding lipoprotein from *E*. *faecium*; SagA; major secreted antigen; VRE, vancomycin resistant enterococci.

### Extrapolation from the transcriptomic data from *E*. *faecalis* in *E*. *faecium*

The transcriptomic data were obtained from the experiments of Muller et al. [22], in which the differences in expression of 368 *E*. *faecalis* proteins was analyzed in a mouse peritonitis model, allowing the identification of a set of 211 up-regulated proteins under infection conditions. Among these up-regulated proteins we analyzed the 18 that corresponded to surface related proteins (e.i. membrane, cell wall associated, extracellular and lipoproteins). The extrapolation of these data in the closely related species *E*. *faecium* was made by the protein BLAST tool (http://blast.st-va.ncbi.nlm.nih.gov/Blast.cgi) comparing these 18 proteins against the sequences available for *E*. *faecium* species (taxid:1352).

### General molecular methods

PCR was performed with Phusion highfidelity DNApolymerase (Finnzymes) and using as template the genomic DNA of the *E*. *feaecium* E155. The primers used are listed in Table 2. PCR products and plasmids were purified using the NucleoSpin Gel and PCR Clean-up and NucleoSpin plasmid kit (Macherey-Nagel). Restriction enzymes and T4 DNA ligase were purchased from Promega and used as recommended by the manufacturer. Genomic DNA extraction and other standard techniques were carried out as described by Sambrook et al. [23].

**Table 2:** Primers used in this study.

Primer name	5´-3´sequence ^+^	Restriction site
**AdcA**_**fm**_ **-5-BamHI**	aggcGGATCCTCGAATGATAAAGATGGAAAAT	BamH I
**AdcA**_**fm**_ **-3-PstI**	aggcCTGCAGTTAATGAGCCATCATTTCTTGA	Pst I
**PsaA**_**fm**_ **-5-BamHI-2**	aggcGGATCCAAAGATACAGTGGCTTCGAACGA	BamH I
**PsaA**_**fm**_ **-3-PstI**	aggcCTGCAGTTATTTCGAAAGGCCTTCAGCA	Pst I

+ Bases in lowercase letters are not complementary to the target sequence. Underlined bases correspond to restriction sites.

### Construction of *E*. *coli* strains M15/pQE30AdcA_fm_ and M15/pQE30PsaA_fm_

The proteins were recombinantly expressed to raise antibodies against the different antigens. The respective genes (EFF33485 and EFF33471) were amplified without including the sequence corresponding to the signal peptide using primers listed in Table 2. The amplicons where then digested with the corresponding restriction enzymes and inserted downstream of the IPTG (Isopropyl β-D-1-thiogalactopyranoside)-inducible promoter into the pQE30 expression vector (QIAexpressionist kit; Qiagen) to obtain an N-terminal His6-tagged recombinant protein. The resulting construct was electroporated into the *E*. *coli* M15pRep4, creating the different M15/pQE30protein strains (see Table 1). Recombinant proteins were overproduced and purified under denaturing conditions using the Protino Ni-NTA Agarose (Macherey-Nagel) resin, following the manufactures instructions. Finally, the purified recombinant proteins were desalted by diafiltration using the Amicon Ultra-15 Centrifugal Filter Units of 3KDa (Merck-Millipore).

### Mass-spectrometry analyses

The identity of the recombinant proteins after affinity purification was performed by mass spectrometry. Overnight tryptic digestion of the obtained proteins was done as described elsewhere [14]. Briefly, after SDS-PAGE and Coomassie blue staining, the protein-containing regions (bands) were excised, and washed twice with ultrapure water and once with acetonitrile/50 mM ammonium bicarbonate (1: 1, v/v). Samples were stirred for 15 min and vacuum-dried for 30 min. In-gel digestion of the excised protein bands was carried out using 0.5 μg trypsin (Promega), incubating overnight at 37°C. Trypsin-cleaved samples were desalted and concentrated on a tipmicroC18 Omix (Agilent) before nano-liquid chromatogramphy nanoLC-MS/MS analysis. The chromatography step was performed on a nano-LC system (Prominence, Shimadzu). Peptides were concentrated on a Zorbax 5x0.3mm C18 precolumn (Agilent) and separated onto a Zorbax 150x75μm C18 column (Agilent). Mobile phases consisted of 0.1% trifluoroacetic acid, 99.9% water (v/v) (A) and 0.1% trifluoroacetic acid, 20% water in 79.9% ACN (v/v/v) (B). The nanoflow rate was set at 300 nl/min, and the gradient profile was as follows: constant 7% B for 5 min, from 7 to 70% B in 183 min, from 70 to 100% B in 5 min, and return to 7% B. The 300 nl/min volume of the peptide solution was mixed with 1.2 μL/min volumes of solutions of 5mg/ml CHCA matrix prepared in a diluant solution of 50% ACN with 0.1% TFA. Twenty nine second fractions were spotted by an AccuSpot spotter (Shimadzu) on a stainless steel Opti-TOF 384 targets. MS experiments were performed on an AB SCIEX 5800 proteomics analyser equipped with TOF ion optics and OptiBeam on-axis laser irradiation with a 1000 Hz repetition rate. The resulting fragmentation patterns were used to determine the sequences of the peptides. Database searching was performed using the mascot 2.3.02 program (Matrix Science). A database corresponding to an updated compilation download from the NCBI database was used with *E*. *faecium* as selected species (including 169 998 entries). The variable modifications allowed were as follows: C-Carbamidomethyl, K-acetylation, methionine oxidation, and dioxidation. Trypsin was selected as the enzyme, with three miscleavages also allowed. Mass accuracy was set to 200 p.p.m. and 0.6 Da for MS and MS/MS modes, respectively.

### Measurement of protein specific IgG titers in polyclonal anti-protein sera

Total IgG concentration was determined for each polyclonal anti-protein sera (i.e. pre-immune serum, test-serum and anti-serum) with the Easy-Titer Rabbit IgG Assay kit (Thermo Scientific) according to the manufacturer’s instructions and adjusted to 1mg/mL. Serum specific IgG titers against AdcA_fm_ and PsaA_fm_ proteins were measured by ELISA as described previously [24]. In brief, Nunc-immuno Maxisorp MicroWell 96 well plates (Thermo Scientific) were coated with 0.20 μg of recombinant proteins AdcA_fm_ or PsaA_fm_ in 0.2M carbonate-bicarbonate coating buffer. Plates were incubated overnight at 4°C and washed three times with washing buffer (WB) (PBS containing 0.05% Tween 20). The plates were blocked with 3% bovine serum albumin (BSA, Applichem GmbH) in PBS at 37°C for 2 hours and washed as described above. Rabbit sera were prepared in twofold serial dilutions (from 1:5 to 1:1,310,720) in PBS supplemented with 3% of BSA, incubated 1 hour at 37°C and washed three times with WB. Alkaline-phosphatase-conjugated anti-rabbit IgG (Sigma-Aldrich) diluted 1:1.000 in PBS supplemented with 3% BSA was used as secondary antibody. After incubation for 1 hour at 37°C the wells were washed four times with WB. Finally, p-nitrophenyl phosphate (Sigma-Aldrich) was used as substrate (1mg/mL in 0.1M glycine, 1mM MgCl_2_, 1mM ZnCl_2_, pH 10.4). After 30min of incubation at room temperature, the absorbance was measured at 405nm on a microtiter plate reader (Multiskan Ascent, Thermo scientific). Each experiment was performed twice at different time-points, and wells were measured in triplicate. Polyclonal anti-protein sera IgG titers were calculated as follows: for each sample, a plot of OD value against the antibody dilution [Log_10_(antibody dilution)] was used to calculate the intercept with the specified cutoff value of each test, and the extrapolated inverse value was used to generate the end point titer [25].

### Opsonophagocytic assay (OPA)

An *in vitro* opsonophagocytic assay (OPA) was performed as described elsewhere [19,21]. Briefly, four components were prepared: (a) baby rabbit serum (Cedarlane Laboratories) absorbed with the target bacterial strain as a source of complement, (b) the different rabbit sera (see Table 1), (c) polymorphonuclear neutrophils (PMNs) freshly prepared from human blood collected from healthy adult volunteers, and (d) the bacterial strains grown to OD_650_ = 0.4 in TSB. Equal volumes of bacterial suspension (2.5x 10^4^μl^-1^), PMNs (2.5x 10^4^μl^-1^), complement source (1.7% and 0.85% final concentration for *E*. *faecalis* and *E*. *faecium* respectively), and either the serum raised against the recombinant proteins or heat-inactivated pre-immune rabbit serum (NRS, as control) were combined and incubated on a rotor rack at 37°C for 90 minutes. After incubation, colony forming units (CFUs) surviving in the tubes with bacteria were quantified by agar culture of serial dilutions. Percentage of killing was assessed as described by Theilacker et al. [26] by comparing the colony counts at 90 min (t90) of a control not containing PMNs (PMNneg) to the colony counts of a tube that contained all four components of the assay using the following formula:

{[(mean CFU PMNneg at t90)-(mean CFU at t90)]/(mean CFU PMNneg at t90)}×100

### Opsonophagocytic inhibition assay (OPIA)

For inhibition studies, rabbit serum raised against the recombinant proteins was diluted 1: 50 and incubated for 60 min at 4°C with the purified recombinant proteins at a final concentration of 100μg/mL, 10μg/mL and 1μg/mL. BSA was used as control without inhibitory activity at a final concentration of 100μg/mL. Subsequently, the respective antibody was used in the OPA as described above. Inhibition assays were performed at serum dilutions yielding 55–65% killing of the inoculum without the addition of the inhibitor. The percentage of inhibition of opsonophagocytic killing was compared to controls without inhibitor.

### Animal model

A mouse bacteremia model was performed to evaluate the passive protection conferred by antibodies raised against the recombinant proteins as describe elsewhere [27,28] with some modifications. In brief, Five female Balb-C mice 6 to 8 weeks-old (Charles River) received intravenously (i.v.) 200 μL of NRS, serum raised against the recombinant proteins or serum raised against recombinant protein SagA as a positive control, 48 and 24h before the challenge. Bacterial inoculum of *E*. *faecium* E155 (6.3 x 10^8^ c.f.u. per mouse) was injected via the tail vein. 24 hours after challenge, mice were humanely euthanized by CO_2_ asphyxiation and colony counts in liver were determined by homogenizing and plating of serial dilutions. Animals were closely monitored for morbidity during the course of the experiment (i.e. at least every 4 hours).

### Statistical Analysis

The software program GraphPad PRISM version 5.00 was used for the statistical analyses. The percentage of organisms killed using immune sera in the opsonophagocytic assay was expressed as geometrical mean ± the standard error of the means. Significance of the bacterial counts in the animal experiment was determined by analysis of variance for multi-group comparisons using log-transformed data, and a Kruskal-Wallis test. A P value of < 0.05 was considered significant.

### Ethics Statement

All animal experiments were performed in compliance with the German animal protection law (TierSchG). The mice were housed and handled in accordance with good animal practice as defined by FELASA and the national animal welfare body GV-SOLAS. The animal welfare committees of the University of Freiburg (Regierungspräsidium Freiburg Az 35/9185.81/G-11/118) approved all animal experiments.

## Results

### Identification and selection of *E*. *faecium* proteins candidates for immunological studies by extrapolation of the transcriptomic data in *E*. *faecalis*

To identify putative up-regulated surface proteins under infection conditions with *E*. *faecium*, the transcriptomic data from the mice peritonitis infection model in the closely related species *E*. *faecalis* was used. Among the 368 proteins studied by the transcriptomic approach in *E*. *faecalis*, 18 resulted to be up-regulated and surface related (e.i. extracellular, lipoproteins, membrane and cell wall associated). We considered that these identified proteins are potential vaccine candidates against infections produced by this species. However, in order to determine which of the identified proteins could be good candidates and be cross-reactive against infections caused by either *E*. *faecalis* or *E*. *faecium*, these proteins were blasted against the *E*. *faecium* sequences available in the NCBI database (taxid:1352). The blast analysis showed that eight of the proteins have significant homologies (Identity > 50% and Query Coverage > 80%) in both species (see Table 3). Among the eight proteins, two showed the highest homology and have been previously described as vaccine candidate in other Gram-positive pathogens. Therefore our studies focused on these two putative surface associated proteins: the 35.6 kDa protein manganese ABC transporter substrate-binding lipoprotein PsaA_fm_ and the 57.4 kDa zinc ABC transporter substrate-binding lipoprotein AdcA_fm_.

**Table 3:** Surface related proteins in *E*. *faecalis* up-regulated in the mice peritonitis model and corresponding BLAST homologies in *E*. *faecium* sequences.

*E*. *faecalis* up regulated proteins			*E*. *faecium* BLAST results^*^				
Accession N°	Name	Mean fold induction[22]	Accession N°	Name	Query cover	Identities	E value
**EF0095**	Lipoprotein^§^	2,7	EFF33494	Esp	36%	46%	5E-04
**EF0163**	Lipoprotein^§^	6,7	WP_002334751	Hypothetical protein	96%	52%	2E-52
**EF0164**	Lipoprotein^§^	6,7	WP_002390048	Hypothetical protein	58%	22%	9E-11
**EF0176**	ABC sugar transport sistem	3,4	EFF34523	Basic membrane lipopprotein	100%	55%	4E-135
**EF0177**	ABC sugar transport sistem	3,4	EFF34523	Basic membrane lipopprotein	100%	60%	4E-143
**EF0361**	Chitinase^§^	46,3	WP_002291042	Chitinase	98%	55%	1E-130
**EF0362**	Chitin binding protein	46,3	EFF34249	Extracellular protein	86%	72%	9E-89
**EF0577**	Lipoprotein	22,1	**EFF33471**	**PsaA**_**fm**_	**100%**	**99%**	**0**
**EF1345**	Sugar ABC transporter	15,4	EFF35767.1	Maltodextrin-binding protein	92%	26%	1E-27
**EF1546**	LysM domain protein	3,7	EFF34140	LysM domain protein	100%	39%	3E-23
**EF1677**	Lipoprotein	9,9	WP_002327802	ABC transporter sugar-binding protein	21%	42%	7E-04
**EF1817**	Serine protease	7,7	WP_002372955	Extracellular metalloprotease family protein	90%	31%	3E-32
**EF1818**	Gelatinase	7,7	EFF35763.1	Phospholipid synthase	2%	53%	4.1
**EF2713**	Cell wall surface anchor family	8,5	EFF34453	Collagen adhesin	22%	37%	1E-03
**EF3193**	LrgB family protein	4,1	EFF33766	LrgB family protein	96%	36%	5E-38
**EF3194**	LrgA family protein	4,1	EFF33767	LrgA family protein	76%	21%	2E-06
**EF3206**	Adhesion lipoprotein	3,1	**EFF33485**	**AdcA**_**fm**_	**100%**	**72%**	**0**
**EF3256**	Pheromone cAD1 Lipoprotein	8,8	ELB00929.1	FMN-binding protein	100%	70%	1E-142

* Standard Protein BLAST against E. faecium sequences in the NCBI (http://blast.st-va.ncbi.nlm.nih.gov/Blast.cgi)

^§^Putative.

PsaA_fm_, manganese ABC transporter substrate-binding lipoprotein; AdcA_fm_, zinc ABC transporter substrate-binding lipoprotein.

### The target proteins induce opsonic antibodies

**Fig 1:**
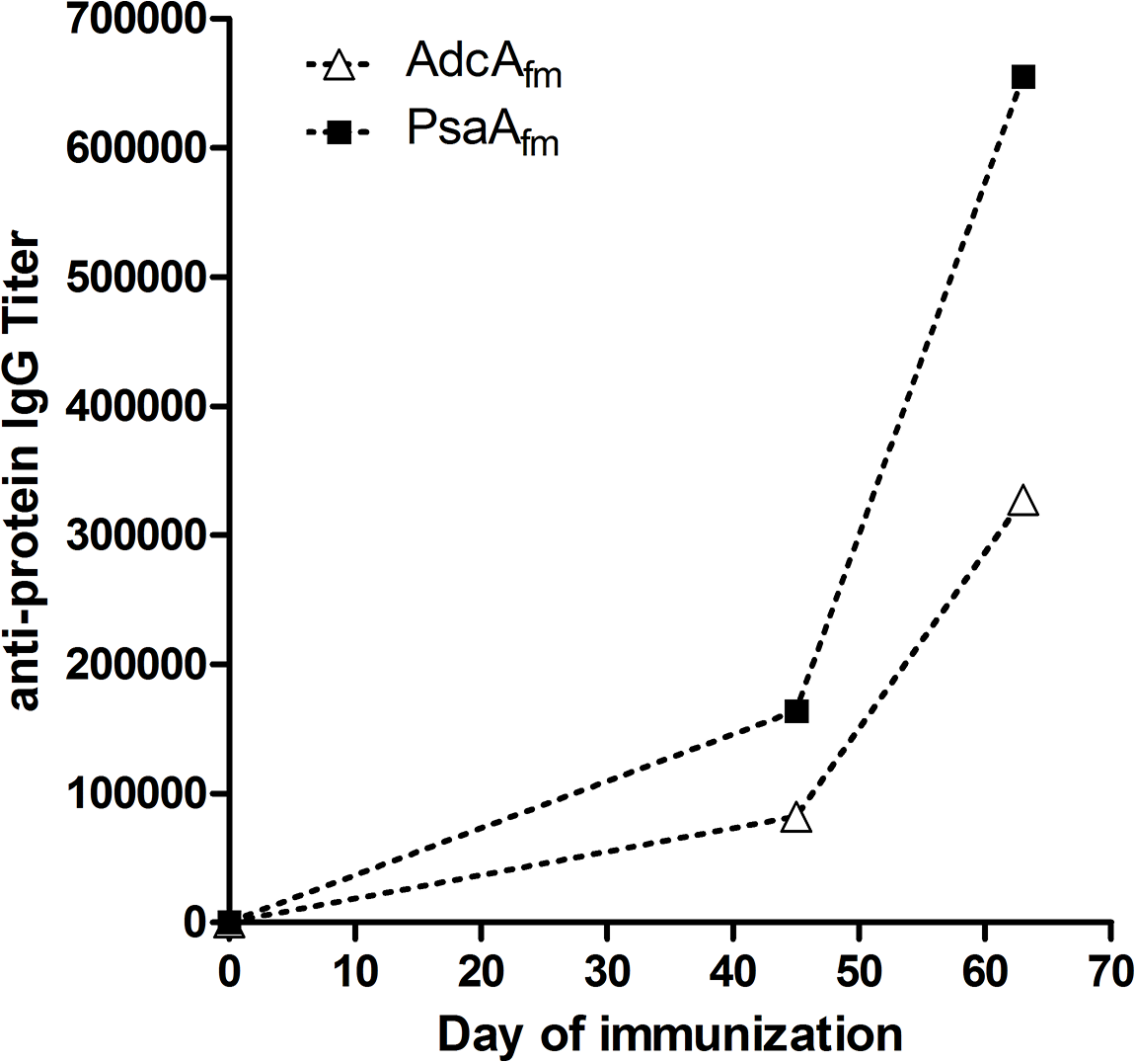
Protein specific IgG antibody titer curves. AdcA_fm_ (black squares) and PsaA_fm_ (white triangles) specific IgG titers of sera raised against the corresponding protein at days 0, 45 and 63 of the immunization procedure.

The genes encoding the two genes EFF33471 and EFF33485, corresponding to the candidate proteins, were amplified by high fidelity PCR without including the sequence corresponding to their signal peptides. These genes were cloned into the pQE30 expression vector and transformed into *E*. *coli M15*. The recombinant proteins were then purified under denaturing conditions. The identity of the proteins was confirmed by LC-MS/MS and their purity was assessed by SDS-PAGE (data not shown). For the production of polyclonal antibodies, New Zeeland white rabbits were immunized with purified proteins and exsanguinated two weeks after the last injection. To confirm that antibodies were generated after immunization against the recombinant proteins, specific IgG titers were measured for each serum at different time points (day 0, 45 and 63) of the immunization procedure. The IgG titers between the terminal bleeding and the pre-immune sera increased 4,000x and 2,000x for the AdcAfm and PsaAfm, respectively (see Fig 1).

**Fig 2:**
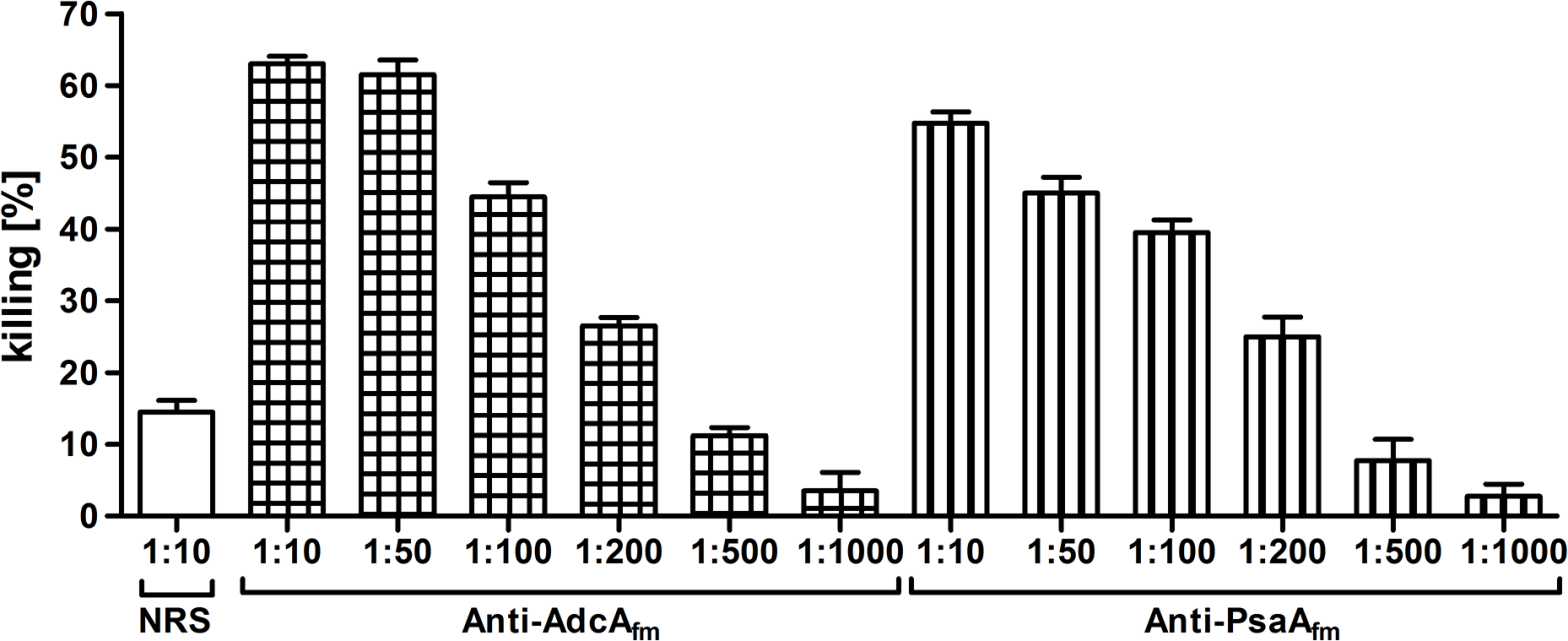
Opsonophagocytic assay used to test the ability to mediate opsonic killing in the strain *E*. *faecium* E155 by antibodies raised against the recombinant proteins at different dilutions. αAdcA_fm_ (square grid) and αPsaA_fm_ (vertical stripes), compare with the activity of pre-immune rabbit serum (white bar). The different sera and the corresponding dilutions used in the OPA are indicated in the abscissa and the % killing in the ordinate. Bars represent the mean of data and the error bars represent the standard error of the mean.

The obtained serum raised against the two different proteins was tested in an opsonophagocytic assay (OPA) against the homologous strain *E*. *faecium* E155 showing that all the proteins were able to induce opsonic antibodies (see Fig 2). To confirm that the opsonic killing observed was mediated by the polyclonal antibodies in the OPA, different concentrations of sera were used to titer out their opsonic activity. The maximum opsonic killing activities of the antibodies at 1:10 dilution were 63% and 55% for the anti-AdcA_fm_ and the anti-PsaA_fm_ sera, respectively. A reduction of killing was observed in a dose dependent fashion using increasingly higher dilutions of sera (see Fig 2).

### The opsonic antibodies are specifically directed against the corresponding recombinant protein

**Fig 3:**
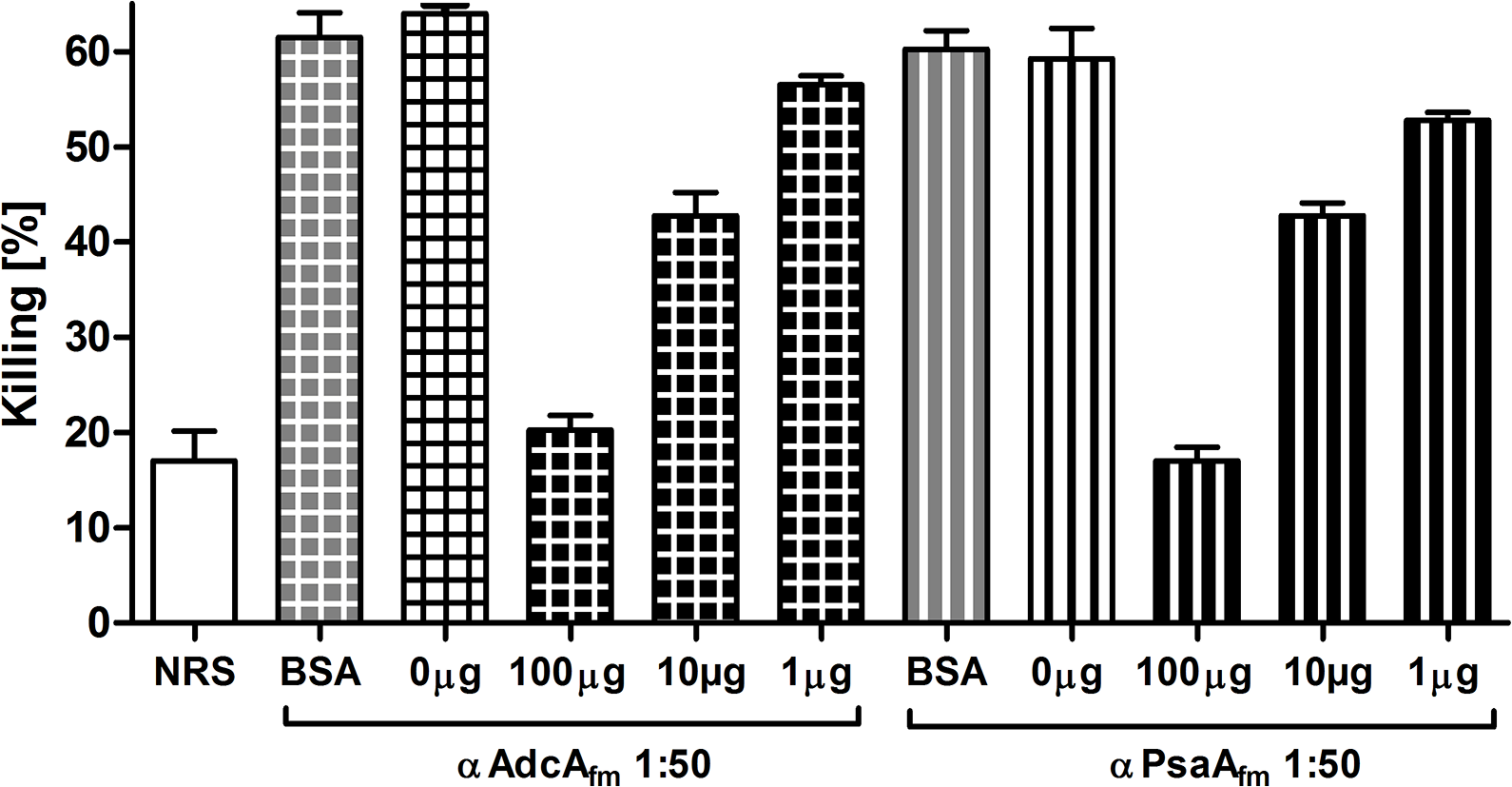
Specificity of antibodies raised against recombinant proteins AdcA_fm_ (square grid) and PsaA_fm_ (vertical stripes). Sera were used at final dilution of 1:50 and the strain tested was *E*. *faecium* E155. Purified recombinant proteins were used as inhibitors at a final concentration of 100μg/mL, 10μg/mL and 1μg/mL. BSA at final concentration of 100μg/mL was used as negative control. The mixtures serum-protein were pre-incubated 1 hour at 4°C prior to OPA. Opsonic killing of the target strain with non-absorbed antibodies was used to assess the reduction of opsonic killing produced by each inhibitor. The corresponding dilutions of antibodies and inhibitor used in the OPA are indicated in the abscissa and the % killing in the ordinate. Bars represent the mean of data and the error bars represent the standard error of the mean.

In order to verify that the antibodies are directed against the corresponding recombinant protein, opsonophagocytic inhibition assays (OPIA) were carried out by pre-incubating the sera with the corresponding recombinant protein in three different concentrations 100, 10 and 1 μg/mL for 1 hour at 4°C. The resulting mixture (anti-protein sera / recombinant protein) was used in an OPA against the *E*. *faecium* E155 strain. A reduction of more than 70% of the opsonic killing was observed in the presence of the highest concentration of recombinant protein tested whereas the control BSA did not show any inhibitory activity at the same concentration. Also, inhibition of opsonic killing decreased in a dose-dependent fashion for both anti-protein sera (see Fig 3).

### Specific and opsonic antibodies are cross-reactive against different *E*. *faecium* and *E*. *faecalis* isolates

**Fig 4:**
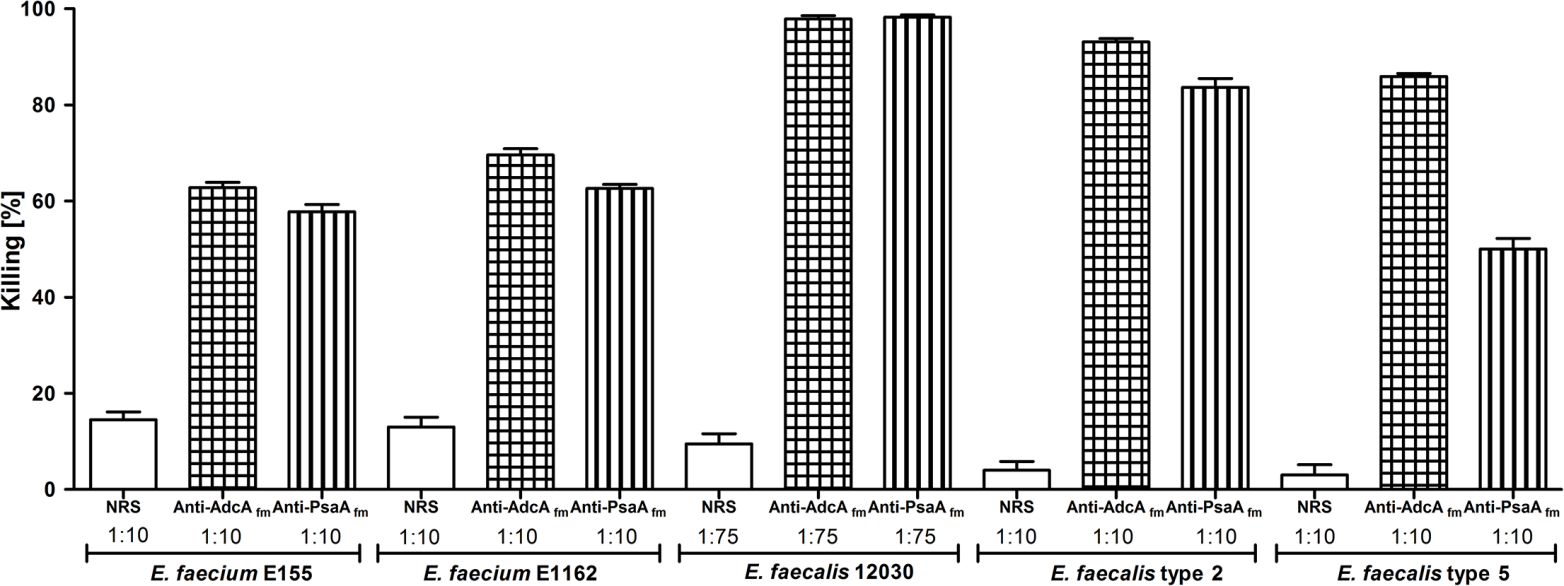
Opsonophagocytic assay used to test the ability to mediate opsonic killing in strains *E*. *faecium* E155, *E*. *faecium* E1162, *E*. *faecalis* 12030, *E*. *faecalis* type 2 and *E*. *faecalis* type 5 by antibodies raised against the recombinant proteins at dilutions between 1:10 and 1:100. AdcA_fm_ (square grid) and PsaA_fm_ (vertical stripes), compared with the activity of the preimune rabbit serum (white bar). The different sera and the corresponding dilutions used in the OPA are indicated in the abscissa and the % killing in the ordinate. Bars represent the mean of data and the error bars represent the standard error of the mean.

To determine if the antibodies directed against the recombinant proteins were able to opsonize other enterococcal strains, serum dilutions between 1:10 and 1:75 were tested in OPAs against *E*. *faecium* E1162 and *E*. *faecalis* strains 12030, type 2 and type 5 [28,29]. The two anti-protein sera were able to opsonize effectively all strains, exhibiting killing above 50% (see Fig 4). A lower opsonophagocytic killing activity was observed for the homologous strain *E*. *faecium* E155 as well as for *E*. *faecalis* strains type 2 and type 5 in comparison with strains *E*. *faecalis* 12030 and *E*. *faecium* E1162.

### Antibodies directed against the different recombinant proteins promote clearance of bacteria in mice livers

**Fig 5:**
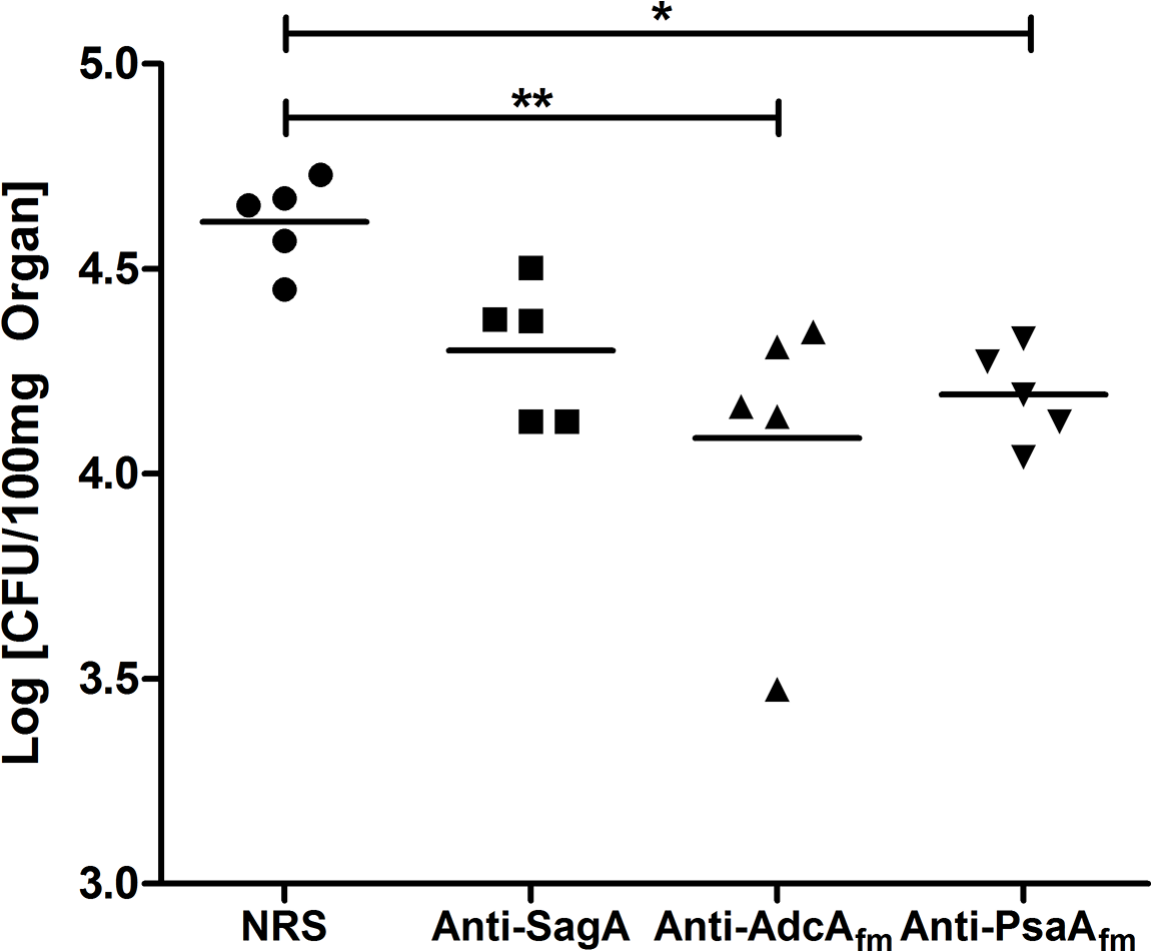
Passive Immunization with the antibodies raised against the recombinant proteins promotes clearance of *E*. *faecium* in mouse liver in comparison with normal rabbit serum. 24 hours after the bacterial challenge mice were sacrificed and livers were removed to assess viable counts. Each point represents the bacterial counts from a single mouse. Bars indicate the median CFU/100 mg of kidney for the group. P value was < 0.05 (*P ≤ 0.05, **P≤ 0.01) for comparison between the animals immunized with the antibodies raised against the recombinant proteins and control animals immunized with NRS determined by analysis of variance for multi-group comparisons using on log-transformed data, and Kruskal-Wallis test.

Mice were passively immunized twice within 48h before bacterial infection to determine if antibodies raised against the recombinant proteins confer protection to mice against bacteremia. Sera raised against the two recombinant proteins significantly reduced *E*. *faecium* E155 colony counts in the livers. The protection conferred by these anti-protein sera seem to be better than the protection conferred by the antibodies raised against the previously reported protein SagA [21]. Immunization with sera raised against protein AdcA_fm_ resulted in lower viable counts (P ≤ 0.01) compared to serum raised against PsaA_fm_ (P ≤ 0.05) and SagA (P > 0.05) (see Fig 5).

## Discussion

Most Gram-positive pathogens possess factors such as anti-phagocytic polysaccharide capsules, surface-associated proteins or toxins that have been studied as potential targets for vaccine development [30]. Most approaches for the discovery of these novel vaccine targets have been done through dissection of the pathogen using biochemical, immunological and microbiological methods [31]. Nevertheless, the arrival of the genome era has dramatically improved the identification of novel vaccine candidates by large-scale high-throughput genomic, transcriptomic, and proteomic analyses [32]. In this work, the identification of some protein vaccine candidates in enterococci has been accomplished by serological proteome analysis (also known as immunomics), i.e. the secreted antigen (SagA), two protein epitopes present on ABC-transporters in *E*. *faecium*, and the Collagen Adhesin in *E*. *faecalis* [21,33,34]. We have recently reported the identification of four additional peptidoglycan associated protein as vaccine candidates (i.e. a low affinity penicillin-binding protein 5, a peptidoglycan-binding protein LysM, a D-alanyl-D-alanine carboxypeptidase and the peptidyl-prolyl cis-trans isomerase) by proteomic analysis [35]. Transcriptomic analyses have been useful tools for the identification of protein antigens involved in pathogenesis allowing the discovery of novel mechanisms of pathogenicity, function of certain proteins and novel therapeutic targets [32,36]. However, to our knowledge, this is the first transcriptomic approach used for the identification and study of putative surface-related proteins as novel vaccine candidates against enterococcal infections.

The two candidate proteins evaluated in this study belong to the family of metal binding lipoproteins (MBL) that act as substrate binding proteins in the ABC transport systems. Some of these MBL have been evaluated as potential vaccine candidates and/or important virulence factors in other Gram-positive pathogens [15,37–44]. The pneumococcal surface antigen A (PsaA) from *S*. *pneumoniae*, which shares 78% of sequence homology with the PsaA_fm_ evaluated in this study, has been widely described as MBL and as an adhesion protein that plays an important role in pneumococcal attachment to the host cell and virulence [40]. PsaA is immunogenic and has been very well studied as a vaccine component against pneumococcal infections [40]. On the other hand, the lipoprotein AdcA_fm_ evaluated in this study shares 64% of sequence homology with the lipoprotein AdcA described in *S*. *pneumoniae*. This protein has been demonstrated to play an essential role as zinc transporter, which is required for proper cell division and for *S*. *pneumoniae* survival during infection, although its potential as vaccine candidate has not been explored yet [39].

The anti-AdcA_fm_ and anti-PsaA_fm_ sera effectively mediated *in vitro* opsonic killing in the parental strain *E*. *faecium E155* as well as other clinically relevant enterococcal strains, i.e. *E*. *faecium* E1162 and *E*. *faecalis* type 2 and type 5 strains, exhibiting killing in a range from 50 to 98 percent. The broad cross-reactivity of the sera showed by antibodies raised against these MBL may effectively overcome the serotype-dependent coverage of polysaccharide-based vaccines.

Surprisingly, we observed lower bacterial killing in OPAs with the homologous strain in comparison with *E*. *faecium* E1162 and a much more pronounced effect with *E*. *faecalis* strain 12030, where the serum concentrations used were seven times lower. As previously explained for other cell wall associated protein vaccine candidates, these differences in opsonophagocytic killing activity may be attributed to hindrance of the target protein by different cell surface determinants, variability in gene expression, protein degradation, and other factors [35,45,46]. Moreover, the presence of capsular polysaccharides in the prototype CPS-C and CPS-D *E*. *faecalis* strains (Type 2 and Type 5) may mask the protein target, explaining the differences observed in the opsonophagocytic activity between these strains.

Further analysis of the sera showed that antibodies specifically recognized the proteins AdcA_fm_ and PsaA_fm_, when opsonophagocytic activity elicited by the anti-protein sera was inhibited by pre-absorption with the target protein. Consistent with opsonophagocytic results, which usually correlate well with *in vivo* immune response and indicates bacteria’s ability to survive in human blood and cause infection [47], prophylactic treatment of mice with the antibodies against AdcA_fm_ and PsaA_fm_ significantly reduced the CFU numbers in the livers. These results demonstrate that passive transfer of the sera confers significant protection against bacteremia in mice after i.v. challenge and suggests that active immunization with these antigens may be feasible. While the levels of protection of mice by passive immunization were statistically significant, there was only about 1 log difference between colony counts in immunized mice versus controls. Therefore, additional experiments using different time-points as well as alternative infection models (e.g. rat endocarditis model) need to be done to strengthen the results. Under the experimental conditions explored in the present study, the two MBL proteins efficiently induced antibodies in rabbits and protected mice against enterococcal infections.

## Conclusion

In summary, we have demonstrated here that i) using transcriptomic data obtained from an *in vivo* model was a successful approach for the identification of novel surface-related proteins that serve as targets for vaccine development (ii) targeting proteins with high homology between closely-related species, i.e. *E*. *faecalis* and *E*. *faecium*, is a good strategy for the identification of novel protein vaccine candidates with a broad coverage among the Enterococcus genus (iii) the proteins PsaAfm and AdcAfm were able to induce specific, opsonic and protective antibodies with a broad cross-reactivity and serotype-independent coverage among the two clinically most important enterococcal species. However, cross-reactive protection studies among other Gram-positive bacteria should be considered as potentially useful for the identification of broadly active vaccine antigens since proteins PsaA_fm_ and AdcA_fm_ shared high homologies with MBL in streptococci, staphylococci and bacillus.
